# The association between metabolic dysfunction-associated fatty liver disease and left ventricular mass index in patients with schizophrenia with long-term hospitalization

**DOI:** 10.3389/fendo.2026.1919402

**Published:** 2026-08-25

**Authors:** Jiahuan Li, Yu Ma, Ying Wang, Qing Wu

**Affiliations:** 1 Department of Psychiatry, Affiliated Psychological Hospital of Anhui Medical University, Hefei, China; 2 Anhui Provincial Mental Health Prevention and Treatment Center; Hefei Fourth People’s Hospital, Hefei, China; 3 School of Mental Health and Psychological Sciences, Anhui Medical University, Hefei, China

**Keywords:** cardiac remodeling, hypertension, left ventricular mass index, metabolic dysfunction-associated fatty liver disease, schizophrenia

## Abstract

**Background:**

Patients with schizophrenia have a high cardiovascular risk, especially long-term hospitalized cases where physical issues are prominent due to prolonged illness, lifestyle restrictions, and antipsychotic use. Metabolic dysfunction-associated fatty liver disease (MAFLD) is an independent cardiovascular risk factor, but its association with cardiac remodeling remains unclear. This study investigates this relationship to support early screening and targeted intervention.

**Methods:**

This study included 430 long-term hospitalized patients with schizophrenia. Echocardiography was used to assess left ventricular mass index (LVMI), and ultrasound to diagnose MAFLD. Demographic, clinical, metabolic, and comorbidity data were collected. Spearman correlation and multiple linear regression were performed to examine the association between MAFLD and LVMI. All analyses were conducted using R, with a two-tailed significance level of α=0.05. Bias-corrected bootstrap mediation analysis was used to assess the mediating role of hypertension and fasting blood glucose.

**Results:**

MAFLD was identified in 231 cases (53.7%) of 430 long-term hospitalized patients with schizophrenia. Compared to patients without MAFLD, those with MAFLD had significantly higher LVMI values (78.79 vs 73.95 g/m², mean difference=4.84, 95%CI: 1.2-8.5, p=0.009). A positive association between MAFLD and LVMI was found using Spearman correlation analysis (r=0.126, p=0.009). Multivariate regression analysis revealed that after adjusting for age, sex, and hypertension, the presence of MAFLD remained positively associated with LVMI (β=3.53, 95%CI: 0.49-6.57, p=0.023). Further mediation analysis indicated that MAFLD had a significant total effect on LVMI (4.178, 95%CI: 1.02-7.34, p=0.010), with hypertension playing a key mediating role in this relationship (indirect effect=1.244, 95%CI: 0.35-2.14, p=0.007), accounting for 29.8% of the total effect. The direct effect of MAFLD on LVMI was not significant after accounting for the mediator (β=2.618, 95%CI: -0.54-5.78, p=0.104).

**Conclusion:**

In this study of long-term hospitalized schizophrenia patients, the total association between MAFLD and LVMI was significant, with a 53.7% prevalence and significantly higher LVMI in affected individuals. Hypertension served as a significant mediator of this relationship, suggesting that the association between MAFLD and cardiac structural remodeling in this population is largely explained by hypertensive pathways. These findings identify MAFLD and hypertension as important metabolic and vascular targets for cardiovascular risk stratification in this population.

## Introduction

Schizophrenia is a long-term mental health condition with a high disability rate and early onset, affecting nearly 1% of the global population (1). It not only reduces the quality of life for patients but also puts a significant strain on healthcare systems and families (2). Based on data from the Global Burden of Disease (GBD), since 1990, the incidence, prevalence, and associated harm of schizophrenia have been steadily increasing (3). Research by Crump C and colleagues shows that the typical lifespan of individuals with schizophrenia is 15 years shorter for males and 12 years shorter for females compared to the general population. The primary cause of death is not the psychiatric symptoms themselves but cardiovascular disease (CVD) (4). Numerous studies have confirmed that the frequency of cardiovascular disease and cardiovascular-related mortality are significantly greater in patients with schizophrenia compared to the overall population (5). Heart failure, as one of the main causes of cardiovascular death, is particularly prominent among schizophrenia patients and often presents with subclinical structural changes and functional impairment of the heart at an early age (6, 7).

In subclinical cardiovascular damage, left ventricular hypertrophy (LVH) is a significant precursor to cardiac mortality and heart failure. An essential imaging indicator for identifying LVH and measuring cardiac remodeling is the left ventricular mass index (LVMI), which is indexed to body surface area. It is well known to be an independent predictor of cardiovascular events and heart failure (8, 9, 10). Notably, patients with schizophrenia have been observed to have a higher risk of LVH and cardiac structural abnormalities even before exhibiting obvious cardiovascular symptoms (11, 12). A cross-sectional study using cardiac magnetic resonance imaging and transthoracic echocardiography showed that individuals with schizophrenia are more prone to structural abnormalities such as thickening of the interventricular septum and posterior wall of the left ventricle, as well as left atrial enlargement (6, 7). Previous research has largely attributed this cardiac remodeling to long-term antipsychotic treatment or disease-related autonomic nervous system dysfunction (13–16). Recent studies indicate that MAFLD can exacerbate left ventricular hypertrophy, promoting cardiac structural remodeling and impacting cardiovascular health, particularly showing a close association with increased LVMI (9). However, neither the underlying mechanisms nor the link between simultaneous MAFLD and LVMI for patients with schizophrenia are fully characterized.

MAFLD, as a central manifestation of metabolic syndrome in the liver, shows a significantly higher prevalence among individuals with schizophrenia (17, 18). This is closely linked to the intrinsic pathophysiological mechanisms of the disease, the adverse metabolic consequences of antipsychotic drugs, and generally unhealthy lifestyles (19, 20). In recent years, MAFLD has been recognized as a systemic metabolic disorder, with increasing attention on its association with CVD (21). In the general population, MAFLD has been shown to be a distinct risk factor for CVD and its subclinical markers (22, 23). Numerous studies have confirmed that MAFLD contributes to cardiac remodeling and elevates cardiovascular risk through mechanisms including promoting long-term inflammation, releasing fibrotic mediators, and inducing insulin resistance. Among those suffering from schizophrenia, the long-term use of antipsychotic drugs combined with poor lifestyle habits further strengthens the link between cardiovascular disease risk and metabolic disturbances (24).

Recent literature has begun to elucidate the pathophysiological links between MAFLD and cardiac remodeling. For example, a study by Li et al. demonstrated that higher fatty liver index was prospectively associated with increased LVMI in two independent general population cohorts (9), supporting the systemic nature of MAFLD-related cardiovascular damage. In patients with severe mental illness, Osimo et al. found that adipose tissue dysfunction and insulin resistance were associated with adverse cardiac remodeling, suggesting shared metabolic pathways (22). Furthermore, recent investigations have confirmed the high prevalence of metabolic complications and their cardiovascular consequences in schizophrenia patients (25, 26). However, most existing research on schizophrenia focuses on cardiovascular diseases or metabolic disorders in isolation, while empirical studies specifically examining the direct relationship between MAFLD and quantitative cardiac structural indicators (such as LVMI) are lacking. Therefore, the aim of this study is to systematically investigate the relationship between MAFLD and left ventricular hypertrophy (as determined by LVMI) in patients with schizophrenia, providing vital clinical imaging data to elucidate the role of MAFLD in cardiac remodeling within this population.

## Materials and methods

### Participants

This cross-sectional study collected data on long-term schizophrenia patients who received treatment at the Fourth People’s Hospital in Hefei City between January 2023 and November 2025. The following were the requirements for inclusion: (1) be between the ages of 18 and 85; (2) have been hospitalized at this institution for more than six months and have been diagnosed with schizophrenia by two psychiatrists using the International Classification of Diseases, 10th Revision (ICD-10); (3) no comorbid diagnosis of other psychiatric disorders; (4) female patients not pregnant or breastfeeding; (5) no severe physical illnesses (such as active infections or malignant tumors) or history of central nervous system diseases; (6) no pre-existing structural heart disease, valvular heart disease, or congenital heart disease. A total of 430 chronic schizophrenia patients meeting these criteria were included, comprising 236 males and 194 females. All participants were of Han ethnicity. During hospitalization, patients received the hospital’s standard diet and participated in appropriately arranged physical activities. The Anhui Provincial Mental Health Prevention and Treatment Center’s Medical Ethics Committee gave its approval for this study, which was carried out strictly in compliance with the Declaration of Helsinki’s tenets.

### Data collection

#### Anthropometric indicators and metabolic diseases

Researchers collected detailed demographic data of schizophrenia patients using a simple patient information form who satisfied the inclusion requirements. The variables included gender, age, education level, disease duration, marital status, height, and weight. Ultrasound imaging was used to assess the presence of hepatic steatosis. Hypertension was defined as a systolic blood pressure ≥140 mmHg and/or a diastolic blood pressure ≥90 mmHg, or current use of antihypertensive medication. Diabetes was defined as fasting blood glucose ≥7.0 mmol/L, or current use of antidiabetic medication. MAFLD was diagnosed based on the presence of hepatic steatosis on ultrasound plus at least one of the following three criteria: (1) overweight/obesity (BMI ≥23 kg/m² in the Asian population), (2) type 2 diabetes mellitus, or (3) evidence of metabolic dysregulation (defined as the presence of at least two of the following: waist circumference ≥90 cm in men or ≥80 cm in women, blood pressure ≥130/85 mmHg or specific drug treatment, plasma triglycerides ≥1.70 mmol/L or specific drug treatment, plasma HDL-C <1.0 mmol/L for men or <1.3 mmol/L for women or specific drug treatment, prediabetes). The body mass index (BMI) was calculated using the formula: BMI = weight (kg)/[height (m)]².

#### Clinical assessment

Two psychiatrists used the Chinese version of the Brief Psychiatric Rating Scale (BPRS) to evaluate the degree of psychotic symptoms (27). A seven-point ranking system, ranging from 1 (not present) to 7 (highly severe), is used for each of the eighteen items on this scale. Higher scores indicate more severe psychotic symptoms. The total score ranges from 18 to 126.

#### Plasma biochemical parameters

Following a period of fasting overnight, blood samples were obtained from each participant between 6:00 AM and 9:00 AM and subsequently analyzed prior to 11:00 AM. The concentrations of blood glucose, total cholesterol (TC), high-density lipoprotein cholesterol (HDL-C), triglycerides (TG), alanine aminotransferase (ALT), aspartate aminotransferase (AST), alkaline phosphatase (ALP), gamma-glutamyl transferase (GGT), creatinine, uric acid, and creatine kinase were quantified utilizing a fully automated biochemical analyzer.

#### Echocardiography

An expert ultrasonography specialist performed each echocardiographic assessment using an ultrasound diagnostic device equipped with electrocardiogram (ECG) synchronization monitoring. Standard two-dimensional echocardiography was applied. The standard imaging planes included parasternal long-axis and short-axis views of the left ventricle, as well as three-chamber (3C), four-chamber (4C), and two-chamber (2C) apical views. The endocardial borders of the left ventricle were clearly delineated, and for each standard plane, dynamic images covering 3 to 5 consecutive cardiac cycles were acquired. To optimize image quality, subjects were told to hold their breath when taking images.

M-mode ultrasound was used to measure the following parameters: aortic diameter, interventricular septal thickness at end-diastole (IVSd), left ventricular internal diameter at end-diastole (LVIDd), anteroposterior left atrial diameter, left ventricular posterior wall thickness at end-diastole (LVPWd), blood flow velocity through the aortic valve, as well as the blood flow velocity through the pulmonary valve.

Under hemodynamically stable conditions, the left ventricular ejection fraction (LVEF) was calculated using the modified Simpson’s biplane method in order to gauge left ventricular systolic function.

Early (E wave) and late (A wave) diastolic blood flow velocities across the mitral valve were measured using pulsed-wave Doppler (reflecting left ventricular diastolic function) and tricuspid valve (reflecting right ventricular diastolic function), and the E/A ratios were calculated accordingly.

LVMI is an indicator used to assess whether the left ventricle is hypertrophied. It is calculated as follows: Left ventricular mass (LVM) is determined using the Devereux formula (also known as the “linear method” (28)): LVM (g) = 0.8 × 1.04 × [(IVSd(cm) + LVIDd(cm) + LVPWd(cm))³ – LVIDd(cm)³] + 0.6 (29). As advised by the American Society of Echocardiography, the body surface area (BSA) is first determined using the Du Bois method based on height and weight: BSA = 0.007184 × height (m)^0.725 × weight (kg)^0.425. Next, the LVMI is calculated by normalizing LVM by BSA: LVMI (g/m²) = LVM (g)/BSA (m²). According to the ASE/EACVI guidelines, an LVMI >115 g/m² for men and >95 g/m² for women is considered elevated (28).

Relative wall thickness (RWT) is an indicator used to examine the left ventricle’s geometric arrangement. It is calculated using the formula RWT = (2 × LVPWd)/LVIDd. An RWT value of 0.42 or higher is considered elevated (28).

Indexed left atrial diameter is used to evaluate whether the left atrium is enlarged. It is defined as the left atrial anteroposterior diameter divided by BSA. The normal range is less than 23 mm/m²; values exceeding this indicate left atrial enlargement.

Two psychiatrists who participated in this study independently reviewed the pertinent data that was taken from medical records in order to decrease data input errors. To assess reproducibility, a random sample of 30 echocardiograms was re-analyzed, yielding intra-observer and inter-observer intraclass correlation coefficients of 0.92 and 0.88 for LVMI measurements, respectively.

### Statistical analysis

R software (version 4.5.1) was used for all statistical analyses in this research. The Shapiro-Wilk test was used to assess the normality of continuous variables. Variables with a normal distribution were presented as mean ± standard deviation (Mean ± SD), and group comparisons were completed using independent samples t-tests. Variables that did not follow a normal distribution were presented as median (interquartile range) [M (IQR)], and the Mann-Whitney U test was employed for group comparisons. Counts and percentages [n (%)] were used to report categorical data, and the chi-square (χ²) test was employed for assessing group differences. Spearman correlation analysis was employed in evaluating the connections between LVMI and various clinical variables. To explore the combined effects of potential factors on LVMI, variables with p < 0.05 in univariate analyses were included based on the Akaike Information Criterion, and, together with findings from previous studies, three sets of independent variables were selected for multiple linear regression analyses. Statistical significance was defined as a two-tailed p-value of less than 0.05. This study used bias-corrected bootstrap methods with 5,000 resamples to estimate total, direct, and indirect effects as well as the proportion mediated (PM) to examine the mediating effects of hypertension and fasting blood glucose on the relationship between MAFLD and LVMI. If a mediation effect’s 95% confidence interval excluded zero, it was considered statistically significant.

## Results

The mean age of the 430 study participants was 51.75 years, with 54.9% being male. MAFLD was identified in 231 instances (53.7%) of them. Compared to the non-MAFLD group, the BMI of the MAFLD group was considerably greater (p < 0.001). Age, gender, concurrent medication use, height, education level, length of the illness, creatinine, creatine kinase, creatine kinase isoenzyme, and chlorpromazine equivalent dose were not substantially different between the two groups. The MAFLD group had significantly higher levels of AST, ALP, ALT, TC, GGT, TG, uric acid, and glucose than the non-MAFLD group (p < 0.05). In addition, there was not a significant distinction between the two groups in either the total BPRS score or any of its five factor sub-scores (**Table 1**).

**Table 1:** Basic clinical characteristics of all participants.

Parameter	Level	Overall	non-MAFLD	MAFLD	p
Parameter		430	199	231	
Age, years		51.75 (12.54)	51.94 (13.19)	51.58 (11.99)	0.767
Sex, n (%)	Female	194 (45.1)	87 (43.7)	107 (46.3)	0.627
	Male	236 (54.9)	112 (56.3)	124 (53.7)	
Marital status, n(%)	Unmarried	202 (47.0)	108 (54.3)	94 (40.7)	0.012
	Married	114 (26.5)	49 (24.6)	65 (28.1)	
	Other	114 (26.5)	42 (21.1)	72 (31.2)	
Hypertension, n(%)	No	305 (70.9)	156 (78.4)	149 (64.5)	0.002
	Yes	125 (29.1)	43 (21.6)	82 (35.5)	
Diabetes, n(%)	No	346 (80.5)	175 (87.9)	171 (74.0)	<0.001
	Yes	84 (19.5)	24 (12.1)	60 (26.0)	
Drug combination therapy, n(%)	No	187 (43.5)	94 (47.2)	93 (40.3)	0.172
	Yes	243 (56.5)	105 (52.8)	138 (59.7)	
Height, m		1.65 [1.60, 1.72]	1.65 [1.60, 1.72]	1.65 [1.60, 1.72]	0.522
Weight, kg		65.00 [60.00, 75.00]	62.00 [55.00, 70.00]	70.00 [65.00, 80.00]	<0.001
BMI, kg/m^2^		24.22 [21.99, 26.97]	22.66 [20.57, 24.44]	25.95 [23.51, 28.24]	<0.001
Education, years		9.00 [8.00, 12.00]	9.00 [8.00, 12.00]	9.00 [8.00, 12.00]	0.697
Systolic pressure, mmHg		120.00 [110.00, 130.00]	120.00 [110.00, 126.00]	120.00 [112.00, 130.00]	0.086
Diastolic pressure, mmHg		80.00 [70.00, 80.00]	80.00 [70.00, 80.00]	80.00 [72.00, 80.00]	0.005
Course of disease, years		23.00 [15.00, 33.00]	24.00 [14.00, 33.00]	22.00 [15.25, 31.50]	0.735
BPRS total points		26.00 [21.00, 33.00]	26.00 [21.50, 33.00]	26.00 [21.00, 33.00]	0.988
Anxiety and depression factor score		4.00 [4.00, 4.00]	4.00 [4.00, 4.00]	4.00 [4.00, 4.00]	0.805
Lack of vitality factor score		8.00 [5.00, 11.00]	8.00 [4.00, 10.50]	8.00 [5.00, 11.00]	0.628
Cognitive factor score		5.00 [4.00, 8.00]	4.00 [4.00, 8.00]	5.00 [4.00, 8.00]	0.187
Activation factor score		3.00 [3.00, 3.00]	3.00 [3.00, 3.00]	3.00 [3.00, 3.00]	0.182
Hostile suspicion factor score		3.00 [3.00, 6.00]	3.00 [3.00, 6.00]	3.00 [3.00, 6.00]	0.449
ALT, U/L		16.00 [10.22, 25.62]	12.00 [8.70, 19.00]	19.00 [13.25, 30.00]	<0.001
AST, U/L		17.65 [14.00, 22.78]	16.60 [13.10, 20.30]	18.50 [15.00, 24.25]	<0.001
ALP, U/L		74.00 [62.00, 87.00]	71.00 [59.50, 86.00]	76.00 [63.00, 87.50]	0.022
GGT, U/L		19.00 [13.00, 30.00]	15.00 [11.50, 21.00]	23.00 [16.00, 38.00]	<0.001
TC, mmol/L		4.09 [3.56, 4.79]	3.98 [3.49, 4.61]	4.19 [3.62, 4.91]	0.011
HDL-C, mmol/L		1.03 [0.86, 1.23]	1.08 [0.92, 1.27]	0.99 [0.82, 1.18]	<0.001
TG, mmol/L		1.30 [0.84, 1.92]	0.97 [0.74, 1.42]	1.58 [1.12, 2.20]	<0.001
Cr,μmol/L		64.33 [55.52, 75.48]	63.90 [55.20, 75.90]	65.20 [55.60, 74.90]	0.595
Uric acid, μmol/L		320.90 [263.00, 379.53]	289.00 [243.80, 338.05]	346.00 [291.95, 405.65]	<0.001
CK,U/L		70.50 [51.00, 102.00]	67.00 [48.00, 104.00]	74.00 [54.50, 100.00]	0.099
Fasting blood glucose, mmol/L		4.88 [4.48, 5.52]	4.70 [4.36, 5.16]	5.06 [4.62, 5.76]	<0.001
CK-MB,U/L		11.00 [9.00, 14.00]	11.00 [9.00, 14.00]	11.00 [8.50, 13.00]	0.329
Equivalent dose of chlorpromazine (mg)		400.00 [250.00, 625.00]	400.00 [227.50, 650.00]	412.50 [300.00, 600.00]	0.743

Data are presented as mean ± standard deviation, median [interquartile range], or n (%). Exploratory analysis, uncorrected for multiple comparisons.

BMI, body mass index; BPRS, brief psychiatric rating scale; ALT, alanine aminotransferase; AST, aspartate transaminase; ALP, alkaline phosphatase; TC, total cholesterol; HDL-C, high-density lipoprotein cholesterol; TG, triglyceride; GGT, glutamyltransferase; CK, creatine kinase; CK-MB, creatine kinase isoenzymes; Cr, creatinine.

**Figure 1:**
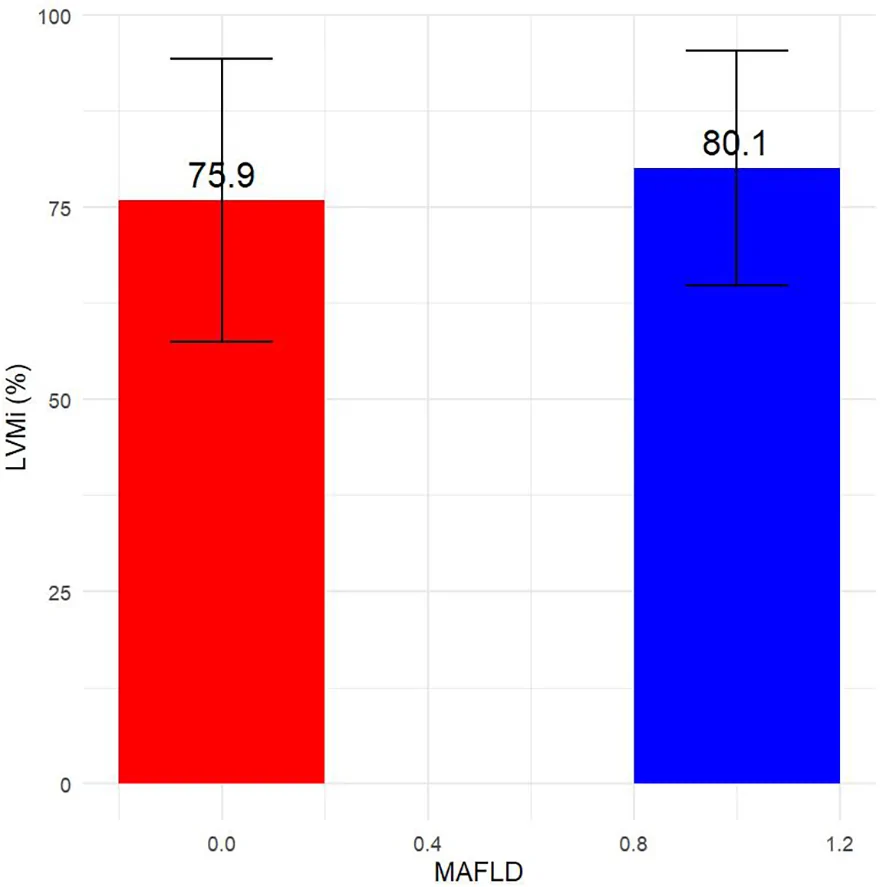
Comparison of LVMI between the schizophrenia group with and without metabolic-related fatty liver disease.

Aortic width, left ventricular anteroposterior diameter, mitral valve E wave, aortic valve, pulmonary valve, indexed left atrial diameter, LVEF, and mitral valve E/A ratio did not vary significantly between patients with schizophrenia who suffered from MAFLD and those who did not (**Table 2**). Yet, there were notable distinctions between the two groups in the mitral valve A wave, left atrial anteroposterior diameter, interventricular septal thickness, left ventricular posterior wall thickness, tricuspid valve E wave, tricuspid valve A wave, RWT, and tricuspid valve E/A ratio. Notably, LVMI, as a cardiac response indicator of metabolic toxicity related to MAFLD, showed a significant difference in schizophrenia patients with MAFLD (p < 0.05). **Figure 1** is a bar chart showing that, among patients with schizophrenia, those with MAFLD have significantly elevated LVMI compared to those without MAFLD (p < 0.05).

**Table 2:** Ultrasound echocardiographic characteristics of all participants.

Parameter	Overall	non-MAFLD	MAFLD	p
Aortic width, mm	28.00 [27.00, 30.00]	28.00 [26.00, 31.00]	29.00 [27.00, 30.00]	0.399
left atrium diameter, mm	31.00 [29.00, 34.00]	31.00 [29.00, 33.00]	32.00 [30.00, 34.00]	<0.001
Anteroposterior diameter of left ventricle, mm	42.00 [40.00, 45.00]	42.00 [40.00, 45.00]	43.00 [40.00, 45.00]	0.184
interventricular septal thickness, mm	10.00 [9.00, 10.00]	9.00 [9.00, 10.00]	10.00 [9.00, 11.00]	<0.001
Posterior wall thickness of the left ventricle, mm	10.00 [9.00, 10.00]	9.00 [9.00, 10.00]	10.00 [9.00, 10.00]	<0.001
Mitral valve E wave, m/s	0.60 [0.51, 0.80]	0.60 [0.56, 0.80]	0.60 [0.50, 0.79]	0.625
mitral A wave, m/s	0.70 [0.60, 0.80]	0.70 [0.60, 0.80]	0.70 [0.60, 0.80]	0.036
Tricuspid valve E wave, m/s	0.50 [0.40, 0.52]	0.50 [0.40, 0.60]	0.40 [0.40, 0.50]	<0.001
Tricuspid valve A wave, m/s	0.40 [0.34, 0.50]	0.40 [0.30, 0.50]	0.42 [0.40, 0.50]	<0.001
Systolic peak velocity of the aortic valve, m/s	1.10 [1.00, 1.20]	1.10 [1.00, 1.20]	1.10 [1.00, 1.20]	0.221
Systolic peak velocity of pulmonary valve, m/s	0.80 [0.70, 0.90]	0.80 [0.70, 0.90]	0.80 [0.70, 0.90]	0.11
Indexation of left atrial diameter, mm/m²	18.19 [16.72, 19.46]	18.37 [17.11, 19.68]	17.93 [16.56, 19.37]	0.074
LVEF, %	62.00 [56.00, 66.00]	62.00 [56.00, 67.00]	62.00 [58.00, 65.00]	0.61
LVMI, g/m^2^	76.89 [67.43, 86.86]	73.95 [64.55, 86.38]	78.79 [70.29, 87.20]	0.009
RWT	0.45 [0.42, 0.50]	0.44 [0.40, 0.48]	0.47 [0.43, 0.51]	<0.001
Mitral valve EA ratio	0.83 [0.71, 1.29]	0.86 [0.71, 1.33]	0.80 [0.71, 1.20]	0.067
Tricuspid valve EA ratio	1.25 [0.80, 1.46]	1.33 [0.98, 1.50]	1.07 [0.75, 1.33]	<0.001

Data are presented as mean ± standard deviation, median [interquartile range], or n (%).

LVMI, left ventricular mass index; RWT, relative wall thickness; LVEF, left ventricular ejection fraction.

**Table 3** presents the correlations between potential risk factors and LVMI. The results of the Spearman correlation study showed that age, hypertension, disease duration, glucose levels, MAFLD, ALP, diabetes, gender, and GGT are positively correlated with LVMI in patients. Specifically, the Spearman correlation coefficient between the presence of MAFLD and LVMI was r = 0.126, p < 0.05.

**Table 3:** Correlation analysis of potential risk factors for LVMI.

Parameter	r	p
Age	0.29	<0.001
Sex	0.111	0.022
Marital status	0.069	0.15
Hypertension	0.259	<0.001
Diabetes	0.111	0.021
Drug combination therapy	-0.051	0.291
Height	0.02	0.681
Weight	0.077	0.11
BMI	0.053	0.274
Education	-0.078	0.105
Systolic pressure	0.083	0.086
Diastolic pressure	0.092	0.058
Course of disease	0.177	<0.001
BPRS total points	-0.011	0.823
Anxiety and depression factor score	0.047	0.329
Lack of vitality factor score	0.044	0.364
Cognitive factor score	-0.046	0.34
Activation factor score	-0.032	0.514
Hostile suspicion factor score	-0.044	0.361
ALT	0.059	0.221
AST	0.026	0.59
ALP	0.119	0.013
GGT	0.102	0.035
TC	0.04	0.412
HDL-C	-0.041	0.395
TG	0.053	0.274
Cr	-0.008	0.872
Uric acid	0.042	0.381
CK	0.043	0.374
Fasting blood glucose	0.144	0.003
CK-MB	-0.042	0.381
MAFLD	0.126	0.009
Equivalent dose of chlorpromazine	0.107	0.132

Exploratory analysis, uncorrected for multiple comparisons. LVEF, left ventricular ejection fraction; LVMI, left ventricular mass index; RWT, relative wall thickness.

**Figure 2:**
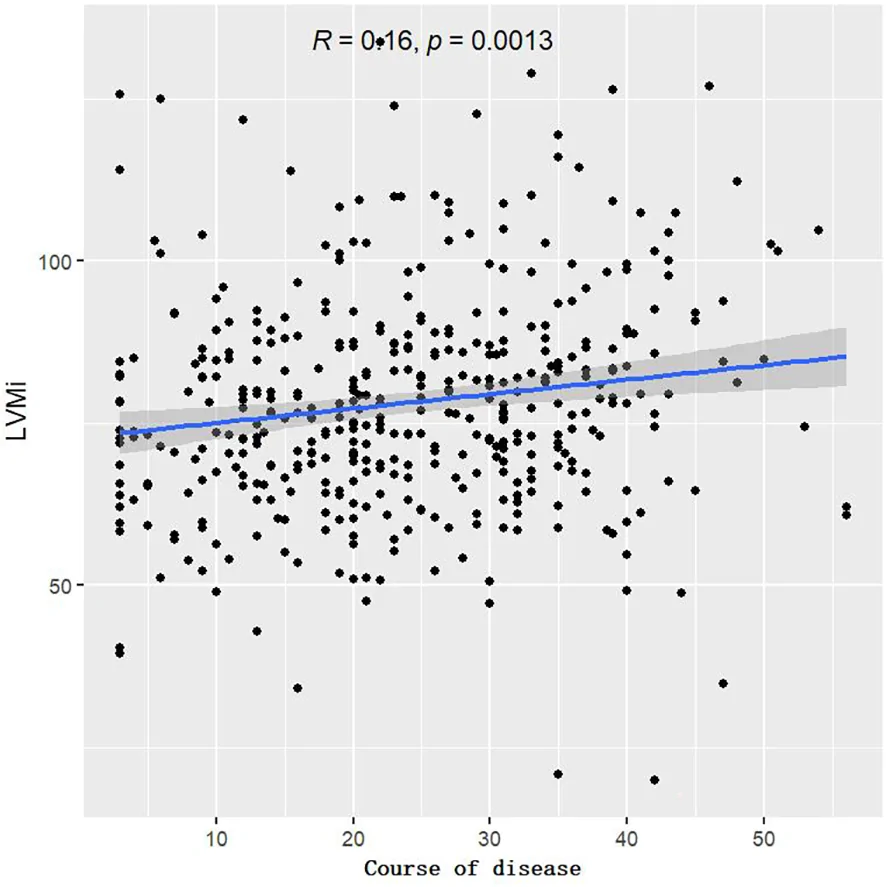
Scatter plot of LVMI versus total BPRS score.

**Figure 2** shows scatter plots illustrating the relationships between clinical features and cardiac LVMI in individuals with schizophrenia. Cardiac LVMI is significantly positively correlated with age (r = 0.27, p < 0.001) and disease duration (r = 0.16, p = 0.001), but not with the total BPRS score (r = –0.031, p = 0.52).

**Figure 3:**
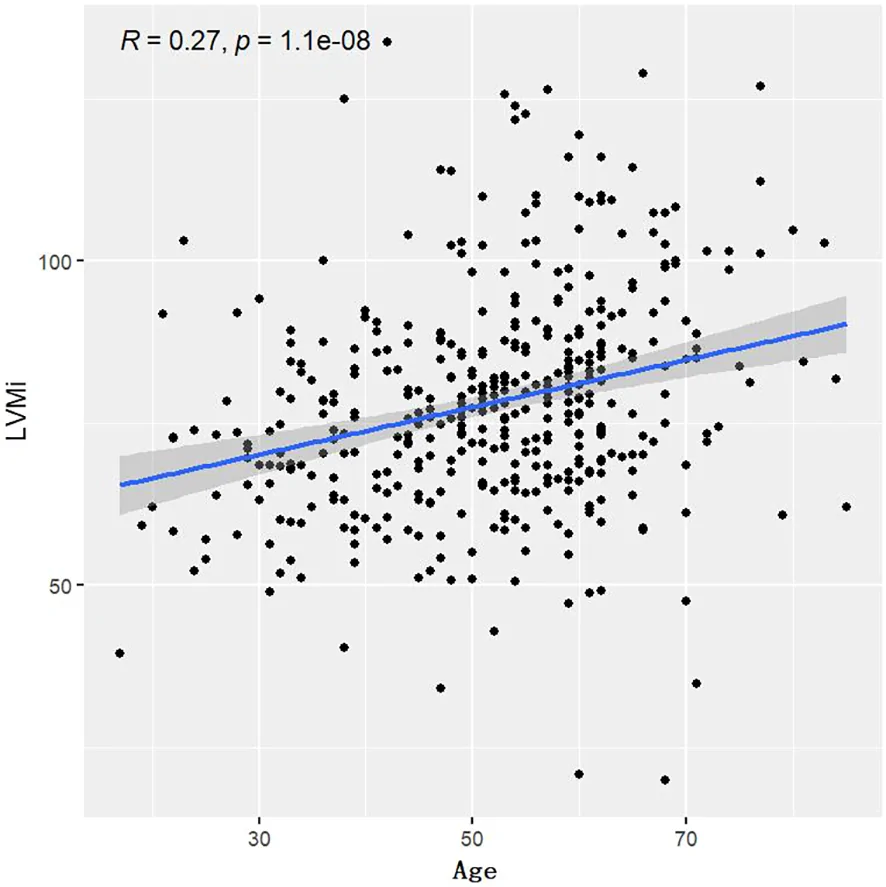
Path coefficient diagram between hypertension, blood glucose levels, MAFLD, and LVMI.

**Table 4** presents the results of the multiple linear regression analysis. Independent variables were selected based on univariate associations (p < 0.05), the Akaike Information Criterion, and prior research. After controlling for sex and age (Model 1), MAFLD was positively associated with LVMI (β = 4.44, p = 0.004). Further adjustments for hypertension (Model 2) showed that MAFLD remained significantly associated with LVMI (β = 3.53, p = 0.023). When diabetes was additionally included (Model 3), the association remained significant (β = 3.41, p = 0.031). However, in the mediation analysis model that simultaneously included hypertension and fasting blood glucose as mediators (**Figure 3**, **Table 5**), the direct effect of MAFLD on LVMI was no longer statistically significant (β = 2.618, 95% CI: -0.540 to 5.776, p = 0.104), suggesting that traditional regression models may have overestimated the independent effect of MAFLD by not accounting for the mediating role of hypertension.

**Table 4:** Regression model of cardiac LVMI in schizophrenia patients.

Parameter	β	SE	p	95% CI	
				Lower	Upper
Model 1					
Sex	4.639	1.557	0.003	1.586	7.691
Age	0.385	0.062	<0.001	0.264	0.506
MAFLD	4.438	1.548	0.004	1.404	7.471
Model 2					
Sex	4.590	1.537	0.003	1.577	7.603
Age	0.308	0.065	<0.001	0.180	0.435
MAFLD	3.531	1.549	0.023	0.494	6.568
Hypertension	6.319	1.804	0.001	2.783	9.856
Model 3					
Sex	4.568	1.539	0.003	1.550	7.585
Age	0.304	0.066	<0.001	0.175	0.432
MAFLD	3.411	1.576	0.031	0.322	6.500
Hypertension	6.291	1.807	<0.001	2.748	9.833
Diabetes	0.256	0.598	0.668	-0.916	1.428

Model 1 adjusted for Sex, Age.

Model 2 adjusted for Sex, Age, Hypertension.

Model 3 adjusted for Sex, Age, Hypertension, Diabetes.

Unstandardized β coefficients are shown.

MAFLD, Metabolic dysfunction-associated fatty liver disease.

**Table 5:** Decomposition of total effect, direct effect, and mediation effects.

Effect	Effect size	Standard error	Lower confidence limit	Upper confidence limit	p	Proportion of Effect
Total Effect	4.178	1.616	1.011	7.345	0.010	100%
Direct Effect	2.618	1.611	-0.540	5.776	0.104	62.7%
Total Mediation Effect	1.560	0.547	0.488	2.632	0.004	37.3%
Mediation Effect (Hypertension)	1.244	0.458	0.346	2.142	0.007	29.8%
Mediation Effect (Fasting Blood Glucose)	0.315	0.300	-0.273	0.903	0.292	7.54%

Bias-corrected bootstrap with 5,000 resamples.

To investigate the underlying mechanism by which MAFLD exerts a significant positive effect on LVMI, this study further incorporated hypertension and fasting blood glucose as mediating variables into the regression model for analysis. **Figure 3** presents the path coefficients among MAFLD, hypertension, fasting blood glucose, and LVMI (***p < 0.001). The model fit indices were excellent (CFI = 0.968, RMSEA = 0.052, SRMR = 0.041), indicating a good model fit. The results showed a significant positive association between the presence of MAFLD and LVMI levels in patients with schizophrenia, with a total effect value of 4.178. Among the mediators, hypertension had the most significant mediating effect: MAFLD significantly positively predicted hypertension (β = 0.286, p < 0.001), and hypertension significantly positively predicted LVMI (β = 0.198, p = 0.014). Bootstrap 95% confidence interval analysis confirmed that the indirect effect of MAFLD on LVMI through hypertension was significant (indirect effect = 1.244, 95% CI: [0.346, 2.142]), accounting for 29.8% of the total effect. In contrast, the mediating effect of fasting blood glucose was not significant; MAFLD’s prediction of fasting blood glucose was not significant (β = 0.102, p = 0.188), nor was fasting blood glucose’s prediction of LVMI (β = 0.045, p = 0.598). Correspondingly, the indirect effect of MAFLD on LVMI through fasting blood glucose was not significant (indirect effect = 0.315, 95% CI: [-0.273, 0.903]), accounting for 7.54%. The direct effect of MAFLD on LVMI also became non-significant after controlling for hypertension and fasting blood glucose (direct effect = 2.618, 95% CI: [-0.540, 5.776]), accounting for 62.7%. In summary, there is a significant positive total association between MAFLD and LVMI levels in patients with schizophrenia, which is primarily mediated through hypertension.

## Discussion

This study systematically investigated the relationship between MAFLD and LVMI in patients with schizophrenia. The results demonstrated that individuals with MAFLD had significantly higher LVMI levels compared to those without MAFLD. Hypertension was identified as a key intermediary factor in this association. In traditional regression models adjusting for age, sex, and hypertension, MAFLD remained associated with elevated LVMI. However, mediation analysis revealed that the direct effect of MAFLD on LVMI became non-significant after accounting for the mediating pathway through hypertension. These findings, for the first time, establish that the relationship between MAFLD and cardiac structural remodeling in this population is largely mediated by hypertension, providing new evidence to explain the pathological mechanisms underlying their high cardiovascular disease risk.

This study is the first to investigate the relationship between MAFLD and cardiac structural indicators, specifically LVMI, in patients with schizophrenia. This finding aligns with previous literature reporting a connection between improper metabolism and cardiac remodeling among people with bipolar disorder (30), further confirming the common role of metabolic dysregulation in cardiac structural damage among patients with severe mental disorders. LVMI has been universally validated as an independent predictor of heart failure and death from cardiovascular disease as a crucial indicator of left ventricular hypertrophy (9). From a pathological perspective, left ventricular hypertrophy leads to reduced coronary blood flow reserve, which in turn causes subendocardial ischemia, loss of myocardial cells, and interstitial fibrosis, ultimately exacerbating cardiovascular disease and heart failure (31, 32). Notably, the frequency of comorbid MAFLD in patients with schizophrenia is significantly greater than that of the overall population (33). Being a recognized independent risk factor for cardiovascular disease (34), MAFLD can increase cardiac workload by elevating blood pressure and accelerating heart rate, thereby promoting an increase in LVMI (35, 36). Research by Pao-Huan Chen and colleagues confirmed that the cumulative number of acute psychotic episodes is independently associated with increased LVMI, reflecting that psychotic episodes themselves are important drivers of cardiac structural damage (37). Moreover, various antipsychotic medications not only directly induce disturbances in glucose and lipid metabolism and promote weight gain, thereby exacerbating MAFLD development (38), but also possess pharmacological properties that prolong the QT interval. This synergistically amplifies the cardiovascular damage effects of MAFLD, further increasing the already elevated CVD risk in patients with schizophrenia compared to the general population (39, 40). Therefore, in clinical practice, special attention should be given to monitoring cardiac health in schizophrenia patients with comorbid MAFLD. Incorporating cardiac structural indicators such as LVMI into routine assessment protocols can provide a basis for early intervention.

This study also observed that patients in the MAFLD group had significantly higher body weight, BMI, TC, and TG levels than those in the non-MAFLD group (P < 0.05), which is consistent with previous findings (41). In these studies, obesity and dyslipidemia (particularly elevated TG levels) were identified as the key pathophysiological mechanisms driving cardiomyocyte hypertrophy and increased LVMI; both can directly damage myocardial structure and promote myocardial fibrosis through oxidative stress and inflammatory signaling pathways (42). These results imply that increased LVMI in individuals with schizophrenia is not the result of a single factor, but rather the outcome of the synergistic effects of metabolic abnormalities such as MAFLD, abnormal blood glucose levels, obesity, and dyslipidemia. In order to lower the risk of heart structural damage, patients with schizophrenia who also have co-occurring metabolic diseases should receive individualized treatment.

This study showed a substantial positive correlation (all P < 0.05) between LVMI in patients with schizophrenia and MAFLD, fasting blood glucose levels, and diabetes status. Blood glucose levels and the incidence of diabetes were significantly higher in the MAFLD group compared to the non-MAFLD group, in line with earlier research findings (22), further confirming the comorbidity and synergistic harm of MAFLD and glucose metabolism abnormalities in patients with schizophrenia. From a pathophysiological perspective, hyperglycemia can exacerbate myocardial remodeling by inducing oxidative stress and promoting the accumulation of advanced glycation end products (AGEs) (43). Additionally, as a core component of metabolic syndrome (MetS) (44), it can drive cardiomyocyte hypertrophy by accelerating atherosclerosis and disrupting myocardial energy metabolism, ultimately leading to ventricular hypertrophy and elevated LVMI (45). Notably, patients with schizophrenia possess a predisposition to metabolic disorders; even without the use of any antipsychotic medications, their incidence of insulin resistance is already above the mean population’s. Furthermore, the use of antipsychotic medications further increases the risk of hyperglycemia (46), leading to a significantly higher prevalence of metabolic syndrome components—such as diabetes—in this population (47), which in turn amplifies the promotion of cardiac remodeling.

A key finding of this study is the central mediating role of hypertension in the relationship between MAFLD and LVMI. Hypertension and MAFLD are core components of metabolic syndrome; they frequently coexist and mutually reinforce insulin resistance (IR), creating a vicious cycle (48). Hypertension is a classic risk factor for hypertrophy of the left ventricle; it can induce cardiomyocyte hypertrophy through increased pressure load, ultimately leading to elevated LVMI (49, 50). In patients with schizophrenia, the risk of hypertension is further influenced by both the disease itself and antipsychotic medications (51). Disease-related autonomic dysfunction can directly disrupt blood pressure regulation, while certain antipsychotic drugs further elevate blood pressure by affecting water and sodium metabolism and exacerbating metabolic disturbances. This combined effect significantly amplifies the adverse impacts on MAFLD and LVMI (52). While the association between MAFLD and LVMI remained significant in traditional regression models adjusting for hypertension as a covariate, mediation analysis revealed that hypertension acts as a pathway variable rather than a simple confounder. This distinction is important: it suggests that hypertension is not merely an independent risk factor but lies on the causal pathway from MAFLD to cardiac remodeling. Therefore, in the cardiovascular risk assessment and management of this population, in addition to routine monitoring of blood pressure, blood glucose, and blood lipids, MAFLD should be regarded as an important warning indicator, and hypertension should be viewed as a key modifiable mediator.

Furthermore, hypertension is substantially linked to schizophrenia patients’ cognitive decline. Research has revealed that schizophrenia patients with hypertension exhibit poorer cognitive function (such as executive function, memory, and attention) compared to those with normal blood pressure (53). Cognitive impairment not only leads to poor lifestyle habits but also increases the risk of metabolic syndrome (54). Existing research has confirmed that timely and effective blood pressure control in schizophrenia patients with concomitant hypertension can rapidly reverse hypertension-induced cardiac structural changes, such as myocardial hypertrophy, while simultaneously improving cardiac function-related indicators (55).

This study has several limitations that need to be addressed in future research:

Cross-sectional design and causal inference: As this is a single-center cross-sectional study, a causal relationship between MAFLD and elevated LVMI cannot be established. The mediation analysis, while informative, cannot definitively establish temporal precedence. Future studies should conduct multicenter, large-sample prospective cohort studies to verify this relationship.

Generalizability: The sample was limited to long-term inpatients from a single hospital, which may restrict the findings’ generalizability. The relatively regular lifestyle of hospitalized patients (e.g., diet, exercise) may have mitigated the severity of metabolic disorders to some extent. Future studies should expand the sample to include community-dwelling patients with schizophrenia.

MAFLD diagnosis and severity: This study relied solely on ultrasound for MAFLD diagnosis and did not perform stratified analyses based on disease severity (e.g., simple fatty liver, steatohepatitis, fibrosis/cirrhosis). Future studies should incorporate fibrosis assessment and more objective measures of liver fat content.

Missing confounders: Although the study adjusted for multiple confounding factors, unmeasured potential confounders (such as dietary details, physical activity levels, smoking status, specific inflammatory markers, and sex hormone levels) may still have affected the accuracy of the results.

Echocardiographic limitations: Echocardiographic assessment is influenced by operator experience and acoustic window conditions. Reliance on echocardiography alone, without using more accurate imaging methods like cardiac magnetic resonance, may lead to measurement bias.

Absence of healthy controls: This study did not include a healthy control group, so it is not possible to directly compare the strength of the association between MAFLD and LVMI in schizophrenia patients to the general population.

## Conclusion

This study has demonstrated a significant association between MAFLD and LVMI in patients with long-term inpatient schizophrenia. According to the findings, up to 53.7% of patients with schizophrenia had MAFLD. Patients with concomitant MAFLD showed noticeably higher LVMI levels. Crucially, hypertension was identified as the primary mediator of this relationship, suggesting that the association between MAFLD and cardiac structural remodeling in schizophrenia is largely explained by hypertensive pathways. For patients with schizophrenia, particularly those with comorbid MAFLD, active monitoring and control of blood pressure are critical for preventing left ventricular hypertrophy and improving cardiovascular outcomes. In clinical management, active intervention targeting reversible metabolic risk factors such as high blood pressure, obesity, and insulin resistance are necessary to improve patients’ cardiovascular outcomes and long-term survival rates.

## Data Availability

The raw data supporting the conclusions of this article will be made available by the authors, without undue reservation.

## References

[1] Hasan A Falkai P Lehmann I Gaebel W . Schizophrenia. Deutsches Ärzteblatt Int. (2020) 117(24):412–9. doi: 10.3238/arztebl.2020.0412 32865492 PMC7477695

[2] Jauhar S Johnstone M McKenna PJ . Schizophrenia. Lancet (London England). (2022) 399:473–86. doi: 10.1016/s0140-6736(09)60722-4 35093231

[3] Solmi M Seitidis G Mavridis D Correll CU Dragioti E Guimond S . Incidence, prevalence, and global burden of schizophrenia – data, with critical appraisal, from the Global Burden of Disease (GBD) 2019. Mol Psychiatry. (2023) 28:5319–27. doi: 10.1038/s41380-023-02138-4 37500825

[4] Crump C Winkleby MA Sundquist K Sundquist J . Comorbidities and mortality in persons with schizophrenia: a Swedish national cohort study. Am J Psychiatry. (2013) 170:324–33. doi: 10.1176/appi.ajp.2012.12050599 23318474

[5] Correll CU Solmi M Veronese N Bortolato B Rosson S Santonastaso P . Prevalence, incidence and mortality from cardiovascular disease in patients with pooled and specific severe mental illness: a large-scale meta-analysis of 3,211,768 patients and 113,383,368 controls. World Psychiatry. (2017) 16:163–80. doi: 10.1002/wps.20420 28498599 PMC5428179

[6] Osimo EF Brugger SP de Marvao A Pillinger T Whitehurst T Statton B . Cardiac structure and function in schizophrenia: cardiac magnetic resonance imaging study. Br J Psychiatry. (2020) 217:450–7. doi: 10.1192/bjp.2019.268 31915079 PMC7511899

[7] Korkmaz S Korkmaz H Ozer O Atmaca M . Assessment of left ventricle systolic and diastolic functions in schizophrenia patients. Psychiatry Res. (2016) 240:348–51. doi: 10.1016/j.psychres.2016.04.025 27138830

[8] Nakamura M Sadoshima J . Mechanisms of physiological and pathological cardiac hypertrophy. Nat Rev Cardiol. (2018) 15:387–407. doi: 10.1038/s41569-018-0007-y 29674714

[9] Li X Heiskanen JS Ma H Heianza Y Guo Y Kelly TN . Fatty liver index and left ventricular mass: prospective associations from two independent cohorts. J Hypertens. (2021) 39:961–9. doi: 10.1097/hjh.0000000000002716 33560053

[10] Bluemke DA Kronmal RA Lima JA Liu K Olson J Burke GL . The relationship of left ventricular mass and geometry to incident cardiovascular events: the MESA (Multi-Ethnic Study of Atherosclerosis) study. J Am Coll Cardiol. (2008) 52:2148–55. doi: 10.1016/j.jacc.2008.09.014 19095132 PMC2706368

[11] Svancer P Capek V Skoch A Kopecek M Vochoskova K Fialova M . Longitudinal assessment of ventricular volume trajectories in early-stage schizophrenia: evidence of both enlargement and shrinkage. BMC Psychiatry. (2024) 24:309. doi: 10.1186/s12888-024-05749-5 38658884 PMC11040899

[12] Rodemer I Konrad M Luedde M Kostev K . The association between schizophrenia and cardiovascular diseases: A retrospective cohort study of primary care routine data in Germany. Brain Sci. (2025) 15(9):974. doi: 10.3390/brainsci15090974 41008334 PMC12468226

[13] Volavka J Czobor P Sheitman B Lindenmayer JP Citrome L McEvoy JP . Clozapine, olanzapine, risperidone, and haloperidol in the treatment of patients with chronic schizophrenia and schizoaffective disorder. Am J Psychiatry. (2002) 159:255–62. doi: 10.1176/foc.2.1.59 11823268

[14] Garcia-Leon MA Fuentes-Claramonte P Valiente-Gómez A Natividad C Salgado-Pineda P Gomar JJ . Altered brain responses to specific negative emotions in schizophrenia. NeuroImage Clin. (2021) 32:102894. doi: 10.1016/j.nicl.2021.102894 34911198 PMC8640102

[15] Chow V Yeoh T Ng ACC Pasqualon T Scott E Plater J . Asymptomatic left ventricular dysfunction with long-term clozapine treatment for schizophrenia: a multicentre cross-sectional cohort study. Open Heart. (2014) 1(1):e000030. doi: 10.1136/openhrt-2013-000030 25332789 PMC4195917

[16] Palatini P . Heart rate reduction and cardiovascular outcome in hypertension. J Am Coll Cardiol. (2017) 69:1099–100. doi: 10.1016/j.jacc.2016.09.991 28231939

[17] Lavagnino L Gurguis C Lane S . Risk factors for metabolic and cardiovascular disease in inpatients with severe mental illness. Psychiatry Res. (2021) 304:114148. doi: 10.1016/j.psychres.2021.114148 34365215

[18] Li X Gao Y Wang Y Wang Y Wu Q . Prevalence and influence factors for non-alcoholic fatty liver disease in long-term hospitalized patients with schizophrenia: A cross-sectional retrospective study. Neuropsychiatr Dis Treat. (2023) 19:379–89. doi: 10.2147/ndt.s398385 36846597 PMC9946011

[19] Vancampfort D Stubbs B Mitchell AJ De Hert M Wampers M Ward PB . Risk of metabolic syndrome and its components in people with schizophrenia and related psychotic disorders, bipolar disorder and major depressive disorder: a systematic review and meta-analysis. World Psychiatry. (2015) 14:339–47. doi: 10.1002/wps.20252 26407790 PMC4592657

[20] Tokarski R Barbosa S Pignon B Aouizerate B Andrieu C Andre M . Clinical and inflammatory markers associated with metabolic dysfunction-associated fatty liver disease in a cohort of individuals with chronic schizophrenia (FACE-SZ). J Psychosom Res. (2025) 195:112192. doi: 10.1016/j.jpsychores.2025.112192 40561671

[21] Mantovani A Csermely A Tilg H Byrne CD Targher G . Comparative effects of non-alcoholic fatty liver disease and metabolic dysfunction-associated fatty liver disease on risk of incident cardiovascular events: a meta-analysis of about 13 million individuals. Gut. (2023) 72:1433–6. doi: 10.1136/gutjnl-2022-328224 36002249

[22] Osimo EF Sweeney M de Marvao A Berry A Statton B Perry BI . Adipose tissue dysfunction, inflammation, and insulin resistance: alternative pathways to cardiac remodelling in schizophrenia. A multimodal, case-control study. Transl Psychiatry. (2021) 11:614. doi: 10.1038/s41398-021-01741-9 34873143 PMC8648771

[23] Xu M Xu H Ling YW Liu JJ Song P Fang ZQ . Neutrophil extracellular traps-triggered hepatocellular senescence exacerbates lipotoxicity in non-alcoholic steatohepatitis. J Adv Res. (2025) 79:521–34. doi: 10.1016/j.jare.2025.03.015 40068761 PMC12766193

[24] Nielsen RE Banner J Jensen SE . Cardiovascular disease in patients with severe mental illness. Nat Rev Cardiol. (2021) 18:136–45. doi: 10.1038/s41569-020-00463-7 33128044

[25] Imre O Imre G Mustu M Acat O Kocabas R . Cardiovascular disease markers in schizophrenia during negative symptoms and remission periods. J Clin Med. (2025) 14:2288. doi: 10.3390/jcm14072288 40217736 PMC11989828

[26] Imre O Kirkas A . The relationship between cognitive function and inflammatory-nutritional status in schizophrenia: cognitive impairment is inversely related to PNI and CALLY indices. BMC Psychiatry. (2026) 26:371. doi: 10.1186/s12888-026-08021-0 41896798 PMC13147825

[27] Hofmann AB Schmid HM Jabat M Brackmann N Noboa V Bobes J . Utility and validity of the Brief Psychiatric Rating Scale (BPRS) as a transdiagnostic scale. Psychiatry Res. (2022) 314:114659. doi: 10.1016/j.psychres.2022.114659 35709637

[28] Lang RM Badano LP Mor-Avi V Afilalo J Armstrong A Ernande L . Recommendations for cardiac chamber quantification by echocardiography in adults: an update from the American Society of Echocardiography and the European Association of Cardiovascular Imaging. J Am Soc Echocardiogr. (2015) 28:1–39.e14. doi: 10.1016/j.echo.2014.10.003 25559473

[29] Devereux RB Reichek N . Echocardiographic determination of left ventricular mass in man. Anatomic validation of the method. Circulation. (1977) 55:613–8. doi: 10.1161/01.cir.55.4.613 138494

[30] Chen PH Hsiao CY Chiang SJ Chung KH Tsai SY . Association of lipids and inflammatory markers with left ventricular wall thickness in patients with bipolar disorder. J Affect Disord. (2024) 358:12–8. doi: 10.1016/j.jad.2024.05.020 38705523

[31] Stewart MH Lavie CJ Shah S Englert J Gilliland Y Qamruddin S . Prognostic implications of left ventricular hypertrophy. Prog Cardiovasc Dis. (2018) 61:446–55. doi: 10.1016/j.pcad.2018.11.002 30408469

[32] Shenasa M Shenasa H . Hypertension, left ventricular hypertrophy, and sudden cardiac death. Int J Cardiol. (2017) 237:60–3. doi: 10.1016/j.ijcard.2017.03.002 28285801

[33] Koreki A Mori H Nozaki S Koizumi T Suzuki H Onaya M . Risk of nonalcoholic fatty liver disease in patients with schizophrenia treated with antipsychotic drugs: A cross-sectional study. J Clin Psychopharmacol. (2021) 41:474–7. doi: 10.1097/jcp.0000000000001421 34086626

[34] Adams LA Anstee QM Tilg H Targher G . Non-alcoholic fatty liver disease and its relationship with cardiovascular disease and other extrahepatic diseases. Gut. (2017) 66:1138–53. doi: 10.1136/gutjnl-2017-313884 28314735

[35] Wu LL Bo JH Zheng F Zhang F Chen Q Li YH . Salusin-β in intermediate dorsal motor nucleus of the vagus regulates sympathetic-parasympathetic balance and blood pressure. Biomedicines. (2021) 9(9):1118. doi: 10.3390/biomedicines9091118 34572305 PMC8467440

[36] Ciobanu A Deaconu MV Portelli PG Gheorghe GS Uscoiu G Nanea IT . PP.26.04] Heart rate variability in relation to left ventricular remodeling and nocturnal blood pressure profile in arterial hypertension. J Hypertens. (2017) 35:e306–7. doi: 10.1097/01.hjh.0000523902.83654.ee 33079766

[37] Chen PH Tsai SY Chiang SJ Hsiao CY Lin YK Chung KH . Association between the number of acute episodes and increased cardiac left ventricular mass index in patients diagnosed with schizophrenia. J Psychiatr Res. (2025) 181:681–8. doi: 10.1016/j.jpsychires.2024.12.023 39746228

[38] Ma Q Yang F Ma B Jing W Liu J Guo M . Prevalence of nonalcoholic fatty liver disease in mental disorder inpatients in China: an observational study. Hepatol Int. (2021) 15:127–36. doi: 10.1007/s12072-020-10132-z 33512644 PMC7886739

[39] Liu Q Yuan X Sheng C Cai W Geng X Liu H . Effect of long-term use of antipsychotics on the ventricular repolarization index. BMC Psychiatry. (2024) 24:505. doi: 10.1186/s12888-024-05947-1 39014414 PMC11250928

[40] Ray WA Chung CP Murray KT Hall K Stein CM . Atypical antipsychotic drugs and the risk of sudden cardiac death. N Engl J Med. (2009) 360:225–35. doi: 10.1056/nejmoa0806994 19144938 PMC2713724

[41] Yi W Wu H Fu W Feng H Huang J Li H . Prevalence and risk factors of non-alcoholic fatty liver disease (NAFLD) in non-obese patients with schizophrenia: A retrospective study. Diabetes Metab Syndr Obes. (2024) 17:841–9. doi: 10.2147/dmso.s437811 38406266 PMC10893889

[42] Chen MY Meng XF Han YP Yan JL Xiao C Qian LB . Profile of crosstalk between glucose and lipid metabolic disturbance and diabetic cardiomyopathy: Inflammation and oxidative stress. Front Endocrinol (Lausanne). (2022) 13:983713. doi: 10.3389/fendo.2022.983713 36187088 PMC9521548

[43] Yoshida Y Nguyen NQ Moon EH Rebholz C Skali H Arthur V . A metabolomics study of cardiac dysfunction in hyperglycemia: findings from the atherosclerosis risk in communities (ARIC) study and the hispanic community health study/study of latinos (HCHS/SOL). Diabetes Care. (2025) 48:1589–97. doi: 10.2337/dc25-0730 40729824 PMC12368386

[44] Neeland IJ Lim S Tchernof A Gastaldelli A Rangaswami J Ndumele CE . Metabolic syndrome. Nat Rev Dis Primers. (2024) 10:77. doi: 10.1038/s41572-024-00563-5 39420195

[45] Luo E Wang D Yan G Qiao Y Liu B Hou J . High triglyceride-glucose index is associated with poor prognosis in patients with acute ST-elevation myocardial infarction after percutaneous coronary intervention. Cardiovasc Diabetol. (2019) 18:150. doi: 10.1186/s12933-019-0957-3 31722708 PMC6852896

[46] van Nimwegen LJ Storosum JG Blumer RM Allick G Venema HW de Haan L . Hepatic insulin resistance in antipsychotic naive schizophrenic patients: stable isotope studies of glucose metabolism. J Clin Endocrinol Metab. (2008) 93:572–7. doi: 10.1210/jc.2007-1167 18029467

[47] Chung MS Yang AC Lin YC Lin CN Chang FR Shen SH . Association of altered cardiac autonomic function with psychopathology and metabolic profiles in schizophrenia. Psychiatry Res. (2013) 210:710–5. doi: 10.1016/j.psychres.2013.07.034 23978730

[48] Donati G Stagni B Piscaglia F Venturoli N Morselli-Labate AM Rasciti L . Increased prevalence of fatty liver in arterial hypertensive patients with normal liver enzymes: role of insulin resistance. Gut. (2004) 53:1020–3. doi: 10.1136/gut.2003.027086 15194655 PMC1774102

[49] Cuspidi C Facchetti R Bombelli M Tadic M Sala C Grassi G . High normal blood pressure and left ventricular hypertrophy echocardiographic findings from the PAMELA population. Hypertension. (2019) 73:612–9. doi: 10.1161/hypertensionaha.118.12114 30612493

[50] Tadic M Cuspidi C Marwick TH . Phenotyping the hypertensive heart. Eur Heart J. (2022) 43:3794–810. doi: 10.1093/eurheartj/ehac393 35869979

[51] Grassi G Seravalle G Trevano FQ Dell'Oro R Bolla G Cuspidi C . Neurogenic abnormalities in masked hypertension. Hypertension. (2007) 50:537–42. doi: 10.1161/hypertensionaha.107.092528 17620522

[52] Correll CU Cutler AJ Laliberte F Germain G MacKnight SD Boudreau J . Impact of cariprazine on body weight and blood pressure among adults with bipolar I disorder, schizophrenia, or major depressive disorder in a real-world setting. Ann Gen Psychiatry. (2025) 24:5. doi: 10.1186/s12991-024-00542-w 39871357 PMC11773801

[53] Hagi K Nosaka T Dickinson D Lindenmayer JP Lee J Friedman J . Association between cardiovascular risk factors and cognitive impairment in people with schizophrenia: A systematic review and meta-analysis. JAMA Psychiatry. (2021) 78:510–8. doi: 10.1093/schbul/sby018.885 PMC7931134 33656533

[54] Liu H Huang Z Zhang X He Y Gu S Mo D . Association between lipid metabolism and cognitive function in patients with schizophrenia. Front Psychiatry. (2022) 13:1013698. doi: 10.3389/fpsyt.2022.1013698 36506447 PMC9729695

[55] Jordan AN Fulford J Gooding K Anning C Wilkes L Ball C . Morphological and functional cardiac consequences of rapid hypertension treatment: a cohort study. J Cardiovasc Magn Reson. (2021) 23:122. doi: 10.1186/s12968-021-00805-5 34689818 PMC8543888

